# Evolution of bright colours in animals: worlds of prohibition and oblivion

**DOI:** 10.12688/f1000research.6493.2

**Published:** 2016-03-22

**Authors:** Wladimir J. Alonso

**Affiliations:** 1 Laboratory for Human Evolutionary and Ecological Studies, Department of Genetics and Evolutionary Biology, University of São Paulo, São Paulo, 05508-090, Brazil

**Keywords:** coral reefs, fish, colours, camouflage, signal-transmission, evolution, conspicuousness

## Abstract

Because the ability to hide in plain sight provides a major selective advantage to both prey and predator species, the emergence of the striking colouration of some animal species (such as many coral reef fish) represents an evolutionary conundrum that remains unsolved to date. Here I propose a framework by which conspicuous colours can emerge when the selective pressures for camouflage are relaxed (1) because camouflage is not essential under specific prey/predator conditions or (2) due to the impossibility of reducing the signal-to-background noise in the environment. The first case is found among non-predator-species that possess effective defences against predators (hence a “Carefree World”), such as the strong macaws’ beaks and the flight abilities of hummingbirds. The second case is found in diurnal mobile fish of coral reef communities, which swim in clear waters against highly contrasting and unpredictable background (hence an "Hyper-Visible World”). In those contexts the selective pressures that usually come secondary to camouflage (such as sexual, warning, species recognition or territorial display) are free to drive the evolution of brilliant and diverse colouration. This theoretical framework can also be useful for studying the conditions that allow for conspicuousness in other sensory contexts (acoustic, chemical, electrical, etc.).

## Introduction

The ability to hide in plain sight is a major selective pressure for both prey and predatory species ^1, 2^. Traits that increase an individual’s capability to camouflage with its surrounding environment have likely been under strong selection pressure since vision emerged, having guided, to a great extent, the evolution of visual displays in the animal world. It is in this context that the eye-catching colouration of fish inhabiting coral reefs and other tropical bodies of water has puzzled scientists since the formulation of the natural selection theory ^3– 11^. Their flamboyance of colour patterns seems to not only disregard any pressures to blend in with the environment, but rather suggests the very opposite purpose: to make an individual stand out as much as possible, competing for attention among members of its own species and predators alike.

Alfred Russel Wallace, co-proponent of the natural selection theory, was also the first to put forth a hypothesis that attributed camouflage properties for those bright colour patterns, whereby “ *brilliantly-coloured fishes from warm seas are many of them well concealed when surrounded by the brilliant sea-weeds, corals, sea-anemones, and other marine animals, which make the sea-bottom sometimes resemble a fantastic flower-garden*” ^3^. A similar argument was proposed to explain the great abundance of eye-catching bird species present amid forest canopy backdrops ^12^. In fact bright colours and patterns can indeed work by disrupting contrasting patterns that can make a prey/predator easily recognizable for some species ^2, 13, 14^. Also, animals we perceive as colourful may not be conspicuous under their natural conditions ^13– 15^. But it has been demonstrated that coral reef fish can possess similar -or even more sophisticated- visual capabilities than humans ^6, 11, 16^; therefore perceiving those colours with the same ease with which we do is within the realm of sensorial possibilities to be exploited for predators and potential preys - negating therefore a camouflage possibility in many (if not in most) cases.

Recognizing the conspicuousness of such colour patterns, the 1973 Nobel Prize winner, Konrad Lorenz, proposed a hypothesis which is based on complete denial of the disguise function in that context. Lorenz suggested these dazzling colour patterns would be a robust means of species-recognition in the highly diverse and multi-niche environment of coral reefs, where such distinct signalling patterns would be needed to prevent aggression among non-competitor species ^4^. A problem for this hypothesis, however, is that many colourful fish found in coral reef habitats are not necessarily aggressive or territorial ^8, 17^, and in any case such selective pressure to be visible would need to overcame the generally much more pressing costs related with higher detectability to predators and/or preys.

Consequently, no hypothesis has withstood existing empirical data, leaving this evolutionary puzzle at large ^4, 7– 11^. Here I argue that conspicuous colours can emerge when the selective pressures for camouflage are relaxed either because camouflage is not essential in the face of specific prey/predator conditions, or due to the biological expense of reducing the signal-to-background noise in the environment.

## The “Hyper-Visible World”

Cott, in his seminal 1940's treatise on the function of animal coloration stated that: " *few birds - whatever their coloration - can be expected to harmonize cryptically with surroundings which vary constantly and widely, from moment to moment and from month to month*" ^2^. Interestingly enough (and perhaps due to the difficulties in observation and study of the behaviour of fish in their habitat before the invention of the scuba), Cott did not extended this reasoning to diurnal mobile coral reef fish (in fact he supported the aforementioned Wallace's hypothesis for this ecosystem).

But coral reef habitats impose to diurnal mobile species the challenge of a continuously changing background, arguably more severe than those in terrestrial environments. As opposed to other marine environments and most terrestrial conditions where backgrounds generally consist of sky blues, earth-tones (sand, stones, snow) or vegetation (mainly green and yellow/brown, resulting from chlorophyll and cellulose), coral reefs have a much more diverse range of conspicuous colours – a direct result of pigments used to protect the symbiotic algae from high irradiances ^18^. And, as previously pointed out, this remarkable chromatic diversity is not out of reach of what fish can perceive ^6, 19^.

Thus the evolution of body-colour in diurnal fish that roam coral reef formations are submitted to special conditions, namely: (1) the high clarity of water during daylight hours and (2) the unpredictable visual pattern of the coral habitat itself. These particular conditions are the ones that critically negate the possibility of camouflage for most diurnal mobile animals in such habitats. While some species can change their body colouration in real time as they roam diverse backgrounds, this is a highly sophisticated and demanding biological feature restricted to only a small subset of species ^2, 20^.

This hypothesis may also be understood within a signal-transmission framework, whereby the visual conspicuousness of an individual is directed correlated with the signal (against background noise) intensity. Accordingly, one of the main selective pressures on colour vision of predators and prey is directed to enhance the perception of contrast between object and background ^6, 20^ and, in order to cancel this out, the aim of camouflage is to induce exactly the opposite. In coral reefs, this signal/noise ratio cannot be reduced by diurnal mobile fish under virtually whatever colour pattern that could be chosen to cover their body. Hence, the exceptionally good environment for signal transmission (clear waters) and the unpredictability of the "background noise" (diverse coral reef) for a mobile individual create exceptionally difficult conditions for the reduction of signal-to-noise ratio (hence the term “Hyper-Visible World”).

Spatiotemporal dynamics ^2, 22^ are, therefore, a critical component in this theory: the degree of mobility of diurnal fish in the geography of coral reef habitats plays a pivotal role in the predictability of the background and hence in the evolution of camouflage. If a fish swims past a variety of backdrops, the likelihood of effective camouflage is close to null – any guise is bound to be seen against one or more backgrounds. If, on the other hand, a fish spends most of its time in one location, natural selection can favour pigmentation and morphologies that match that predictable substrate (be it a coral species, type of rock or sand colouration).

This “Hyper-visible world” hypothesis presents a specific and falsifiable ( *sensu* Popper ^23^) prediction: other traits being equal, roaming fish with any degree of visual prominence will endure equivalent predatory pressure (or success) in coral reefs, but not when swimming against a predictable and homogenous background.

Since signalling patterns evolve as a trade-off between predation and other selective pressures ^21^, when predation under varying degrees of visual conspicuousness is similarly efficient, other selective pressures for visual communication that benefit from conspicuousness can evolve without the constraints imposed by the need to camouflage. Those selective pressures range from hostile to friendly signalling. Among conspecifics, for example, signals range from those communicating willingness to engage in dispute over resources to stressing bonding forces for school formation and sexual attraction ^2, 4, 8^. In interspecific interactions, signals may range from warnings of retaliatory weaponry (e.g. aposematism by poisonous fishes) to the marketing of services (e.g. special colours and approaching behaviours of cleaner fishes ^2, 4, 24^).

It is interesting to note, however, that in this “Hyper-visible world”, while selective pressures for conspicuousness are favoured by the transparency of the medium, they are hampered by the complex and colour-rich background of the coral reef – hence the pressure for the “Hyper-unnatural” (i.e., not often found in nature) colour patterns of many reef fish. By the same token, the need for cryptic species to “deceive with perfection” of cryptic species is also exceptionally high, leading to the “Hyper-naturalism” of fish species like the pygmy sea horse or anglerfish, which is more typical of terrestrial environments (where visibility is also usually excellent) than of other marine habitats.

The fact that most birds don't display similarly flamboyant body colouration might be an indication that terrestrial habitats are perhaps not such a “Hyper-visible world” after all (in the sense that there are still plenty occasions for using some concealment – particularly because, as opposed to mobile diurnal coral reef fish, several hours of daylight are usually spent in resting places). But there are notable exceptions to "modest" coloration of birds - and the conditions that allowed some species of birds to overcome the need for camouflage to display dazzling colours, observed in some species, are of a different “world” – one that doesn't care, as we will see next.

## The "Carefree world”

**Figure 1.**
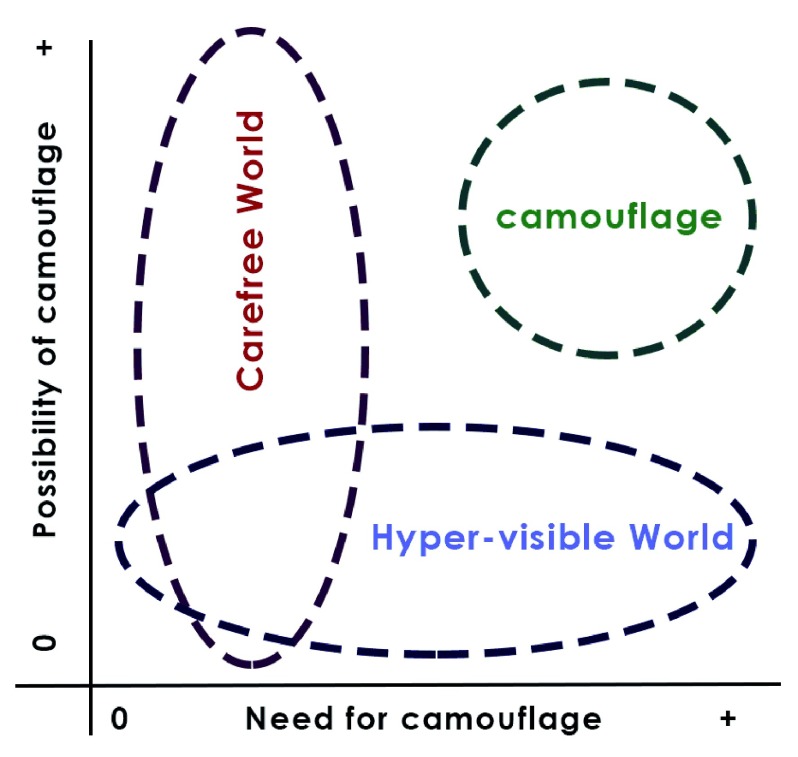
Diagram displaying the landscape of sensorial (y axis) versus ecological (x axis) constrains on colouration that animals can develop to answer the predatory-prey pressure. The “Need for camouflage” axis refers to the selective pressures that drive camouflage. The “Possibility of camouflage” axis springs from the environmental and physiological constraints of the signal transmission between predators and prey. Animals that are under relaxed pressure for camouflage (e.g. like macaws and hummingbirds, which are non-predators and with strong defences against predators) are in the “carefree world” arena. Animals in the “Hyper-visible world” are those that are prevented from developing camouflage (e.g. diurnal mobile fish on the coral reef environment).

The “Hyper-Visible World” hypothesis relies on the notion that camouflage among coral reefs is not an option for many diurnal mobile species. But selection for camouflage can be also relaxed in other contexts. One such case is that of non-predatory species endowed with effective defence mechanisms against predators. The hummingbirds’ speed or the nut-cracking beaks of macaws probably did not evolve first as protections against predation, but are effective in that sense (i.e. became *exaptations* ^25^ for defence), freeing those animals from the need to invest biological capital to visually blend in with their surroundings ^2^. Instead of an impossibility of camouflage found in the “Hyper-visible world”, these birds live in a “carefree world” in which concealment is simply not needed ( Figure 1).

Back to the coral reef conundrum, it has been proposed that diurnal coral reef fish have a visual advantage over their predators as a result of their high visual acuity, coupled with quick access to safe havens among the complex structures of the coral reefs ^6^. If this is the case, they are also living in a “carefree world”, and therefore in the overlapping region of Figure 1.

## Final remarks

Justin Marshall, a specialist in the study of colour vision observed, regarding the dazzling colours found in coral reef, that it *"is almost inconceivable for only one evolutionary force to be behind the colours of such a diverse assemblage* ^8^”. Indeed, should the hypothesis presented here prove accurate, it is paradoxically the very elimination of only one evolutionary force (concealment of potential predators/prey by cryptic colouration) that sets the artistic boldness of several other evolutionary pressures free to draw the magnificent mosaic of colours and shapes found in these marine habitats.

Be it due to prohibition (“hyper-visible world”) or oblivion (“carefree world”) to concealment, once the pressures for camouflage are relaxed, other roles for bright colouration can take over, opening up an assortment of evolutionary possibilities. For instance, we can speculate that signalling in high visual resolution and with conspicuous coloration can promote the genesis of new species through sensory-drive ^21, 26^, a process whereby subtle changes in either colour patterns or in sensory/cognitive biases for attraction to those patterns can lead to the reproductive isolation of part of a population. In this sense, the high resolution of signals coupled with the high productivity of coral reefs might account for the high rates of sympatric speciation observed in these habitats.

Evolutionary possibilities, of course, are not necessarily fulfilled: not only camouflage, but also bright coloration are expected to represent usually costly energetic and evolutionary investments. Therefore, the potential to develop bright coloration due to the lack of selective pressure for camouflage will not necessarily be fulfilled (an obvious example of this in the “Carefree world” can be observed among adult whales or elephants, where the absence of natural predators or need for concealment to obtain food does not imply that they will parade macaw-like coatings). Biological economy presses for neutrally-adaptive colours in those cases (or colours that are a by-product of other functions different from the ones related to visual communication, such as thermal regulation, UV protection, structural, etc).

Finally, animals are multi-sensorial entities: the circumstances that relax the evolutionary pressure for visual concealment ( Figure 1) should be considered analogous to those enabling conspicuousness in other dimensions (e.g., acoustic, olfactory, tactile, electrical). This field may greatly benefit from future research that investigates the evolutionary pathways that open when special conditions stops to impose crypsis. A striking example comes from the acknowledgment of this aspect on the emergence of our own species (the only “who live in the ground and can sing” ^27^). By doing so, the ethnomusicologist and evolutionary musicologist Joseph Jordania recently proposed the intriguing hypothesis that the primordial human evolution was shaped by the incorporation of a set of conspicuous features (like dancing and polyphonic singing to prepare for and trigger a coordinated attack) that turned afraid tree-living monkeys into assertive erect and noisy hunt-stealers from lions ^27^.

Caught existentially in a food web, every time we observe a species (being flamboyant fishes of corals, flashy hummingbirds or noisy humans) that seems to show disregard for the unwritten -but unforgiving- rules of discretion, there is a certainly a fascinating evolutionary conundrum to be solved. Specifically for the case of the coral-reef fish colours that intrigued so many scientists, the framework presented here provides a fresh and testable scientific hypothesis.
