# Supplementary material for: Evolution of bright colours in animals: worlds of prohibition and oblivion
Source: F1000Res. 2016 Mar 22;4:115. Originally published 2015 May 12. [Version 2] doi: 10.12688/f1000research.6493.2 (PMC5089127; doi:10.12688/f1000research.6493.2)
Supplement: Peer review history

# Peer review: Referee response for version 2

**DOI:** 10.5256/f1000research.8965.r16149

**Published:** 2016-10-11

**Authors:** Brett Seymoure

**Affiliations:** 1 Department of Biology, Colorado State University, Fort Collins, CO, USA

In this opinion piece by Alonso, the hypothesis that animals in very colorful environments such as coral reefs, should not evolve cryptic coloration due to the impossible requirements of the myriad colorful backgrounds is proposed. Alonso also hypothesizes that animals that have putatively escaped predation pressures due to exaptations (such as the strong beaks of Macaws and the fast speed of hummingbirds) also will not rely on camouflage for protection. Most of the ideas presented here are hypotheses that have been presented before by Poulton in the late 1800's and Cott in his seminal book on animal coloration published in the 1940's. The field of animal coloration is deserving of a revisit to these ideas, however, I believe the current article needs more logical evidence before it can be accepted as a framework by evolutionary and behavioral ecologists studying components of coloration.

 The article is conveniently broken into two main hypotheses: 1) Hyper-visible World; and 2) Carefree World. The hyper-visible world needs much more support from previous research before being considered further. There are many examples of animals that use camouflage in coral reefs. A major issue with this article is that the author does not make distinctions between background matching and disruptive coloration, both of which are camouflage tactics. This article can be greatly strengthened by including the tradeoffs of background matching and disruptive coloration. Background matching is unlikely to occur in coral reef fish that are highly vagile, however, one would predict that fish that move around in a very heterogenous environment (e.g. a coral reef) would evolve coloration that obstructs the outline of the animal as well as impeding the judgement of speed by a potential predator. The author needs to add to this opinion to include this distinction as well as include this into the figure. 

 Another concern of the hyper-visible world hypothesis is the assumption that animals will evolve camouflage before any other type of coloration (e.g. social and/or sexual signals). Alonso states that the diversity of flamboyant coloration is a Darwinian puzzle since strong selection pressures from predators should have selected against bright, conspicuous coloration. However, Alonso disregards one of Darwin's most influential books, The Descent of Man and Selection in Relation to Sex, in which Darwin describes how gaudy individuals can receive a fitness benefit from attracting mates. Of course, there are now many more hypotheses explaining gaudy appearances in animals ranging from mimicry to social communication. The author needs to address these other hypotheses and explain under what conditions the hyper-visible world will mask these needs for bright coloration. 

 As for the second hypothesis, the Carefree World, this needs much more development and the author must cite empirical research stating that the animals of interest (e.g. the Macaw and hummingbirds) are not under predation pressures. The conclusion that Macaws are exempt from predation due to their strong beaks and claws seems premature without studies testing this. A study by Burger and Gochfeld ^1^ show that Macaws had many anti-predator behaviors that have evolved to reduce predation from eagles and hawks. Furthermore, personal communication with two experts in the field of hummingbird coloration, Melissa Meadows and Richard Simpson, have informed me that hummingbirds are predated upon by mantids and cats. Thus, I am reluctant to agree with Alonso's opinion on the carefree world as it is currently written. It is not beyond reason that some animals may have evolved bright coloration due to a lack in predation, I just think the current argument needs more corroboration. 

 The concluding remarks are welcomed as Alonso includes the discussion of sensory drive and I think that sensory drive is very important in the topics discussed here, however, I don't think that sensory drive is a result of a carefree world or a hyper visible world as there are many examples where predators affect the evolution of coloration in animals with sensory drive (e.g. cichlids and guppies). I suggest that the author includes sensory drive into his hypotheses and I also believe that other forms of coloration such as the many types of mimicry, also deserve discussion. 

 I appreciate the figure that the author provided but I have some reservations. First, the figure appears to say that physiological constraints drive the environment, which is confusing to me. Second, the two axes are different levels of analyses and thus the figure is difficult to interpret. The y-axis is physiological constraints, which is a proximate explanation, while the x-axis is the fitness benefit, which is an ultimate explanation. I am not stating that this is incorrect because I agree with the author that both explanations must be met for the proposed hypotheses to be possible, but much more explanation is needed to allow the reader to understand how the different levels of analyses are included. 

 Lastly, I agree with the previous reviewers in that the optics of the water as well as the visual system of the different viewers needs to be incorporated.

I have read this submission. I believe that I have an appropriate level of expertise to confirm that it is of an acceptable scientific standard, however I have significant reservations, as outlined above.

## References

[1] BurgerJGochfeldM: Parrot behavior at a Rio Manu (Peru) clay lick: temporal patterns, associations, and antipredator responses. *Acta ethologica*.2003;-1(1) : 10.1007/s10211-003-0080-y 10.1007/s10211-003-0080-y

# Response

**Published:** 2016-10-19

**Authors:** Wladimir Alonso

**Affiliations:** Laboratory for Human Evolution Studies, Genetics and Evolutionary Biology, University of São Paulo, Brazil

I thank the reviewer for his comments and stimulating discussion. Nevertheless, I must confess that when I received this feedback I was drawn to the comment that " *Most of the ideas presented here are hypotheses that have been presented before by Poulton in the late 1800*". I would appreciate if the reviewer could provide a more precise citation, as I have consulted Poulton’s 1890’s book “ *The colours of animals, their meaning and use, especially considered in the case of insects*” and could not find anything similar to the ideas presented here. If there are other studies (or some part of that book that I missed) containing those, I would greatly appreciate this information as I went into great length to give credit to past studies.

 The fact that so many brightly colored animals are found in coral reefs has been addressed numerous times since Wallace – but not resolved yet, as discussed by the many authors cited in the introduction of this paper (we can also add this statement from Dr. Gil Rosenthal - one of the top researchers in this field- “ *It’s something we don’t, as scientists, fully understand, or come close to understanding*” and “ *It’s just such a beautiful part of nature that we don’t have an easy explanation for*” http://onlineissues.wherewhenhow.com/publication/?i=100968&ver=html5&p=74). In fact, when I found that Cott formulated an idea similar to the “hypervisible word” for birds (but not for coral reef fish, as he believed -in consonance with Wallace’s  and most current views- that in coral reefs bright colors have a camouflage role) I promptly cited and credited his insight. Therefore, I would be in fact quite surprised if the hypothesis presented in this manuscript has been published before.

 The reviewer also states that “ *the current article needs more logical evidence before it can be accepted as a framework by evolutionary and behavioral ecologists studying components of coloration*”. I am struggling with this suggestion and what was meant by “logical evidence” in this context. I believe (and so do the other reviewers) that the framework presented is a useful one for guiding future research in this area, which in turn may provide evidence to support or refute this conceptual framework. *Evolutionary and behavioral ecologists studying components of coloration* of mobile fish in coral reef now have another biologically sound and falsifiable hypothesis (and an expanded theoretical framework) that can hardly be ignored - at least until (and if) rejected by enough empirical evidence or some biologic flaw be found in this one.

 The reviewer comments that “ *the author does not make distinctions between background matching and disruptive coloration, both of which are camouflage tactics*”. Background matching is hard to achieve for moving background-matching objects - they would need to perfectly match their background and the background would need to be homogenous; whereas disruptive coloration tends to feature too many surface landmarks (e.g. contours and blotches), which provide perfect tracking cues on moving objects (George Lovell, *pers. comm*.. For an illustration of the latter principle, please see this video provided by George Lovell https://youtu.be/HEePyENN1HA). Both mechanisms of attempting crypsis pointed out by the reviewer are therefore not effective for mobile fish in the heterogeneous and unpredictable coral reef environment.

 The reviewer cites Darwin’s explanation for the role of sexual selection in the evolution of bright color. Of course bright coloration can be beneficial for several biological reasons (species recognition, aposematism, aggressiveness - and of course, sexual selection). Such selective pressures are acknowledged in the current manuscript as the driving force behind the evolution of coral reef fish colors. But such coloring is not the point addressed here. This manuscript addresses the conditions that allow coral reef fish (or species like macaws and hummingbirds) to possess extremely bright colors and patterns (in response to those pressures), which are not seen in most other species of birds (and other many ecosystems apart from coral reefs).

 I agree with the reviewer that sensory drive is not a result of either a "Carefree World" or "Hyper-Visible World”. In fact, “sensory drive” is mentioned only as a complementary hypothesis related to the possibility of higher rates of sympatric speciation due to better opportunities of recognition when species are “free” from the demands/possibility of concealment. Moreover, "sensory drive" is not relevant to testing the hyper-visible world hypothesis against Lorenz’s or Wallace’s alternative. Therefore, as interesting as other forms of coloration and sensory drive are, there is not much room to follow the reviewer’s suggestion and expand their discussion.

 Regarding the Carefree World hypothesis, I welcome the presentation of empirical data by the reviewer aimed at challenging it, and I am glad to discuss those challenges. The fact that macaws have many anti-predator behaviors that have evolved to reduce predation from eagles and hawks does not refute the carefree world hypothesis: as anyone who has handled macaws knows, their strong beaks are an extraordinary defense (that most other birds don’t have) against predators - and so the evolutionary pressure for concealment might be relaxed to the point that other selective pressures for conspicuous patterns (sexual, species identification, etc) in terms of body colors (and possibly other features such as their noise vocalizations) have a bigger role than camouflage. But that does not mean that the selection against predators would be eliminated. Similarly, we don’t expect that sexual selection to be absent in animals with camouflage.

 The observation presented by the reviewer regarding hummingbirds is quite interesting - and I believe a good example where the framework I presented can help to understand the evolution of bright colors. The acrobatic and fast flights and take-offs (together with their tiny size) of hummingbirds make them an unworthy target for most predators in most situations - hence enabling them to live in a “Carefree World” most of the time. Nevertheless, as pointed out by the reviewer, Mantis and cats hunt hummingbirds. But the majority of predation is likely to occur when hummingbirds are feeding in flowers (or artificial feeders), or eggs or nesting hatchlings. In the feeding in flowers circumstances, the mechanics involved (with movement, unpredictable background and highly visible medium) a humming bird is impossible to camouflage. Therefore, in those particular instances what they face are actually the challenges of the “Hyper-Visible World”, where camouflage is simply not possible. And (again using the framework presented in figure 1 of the manuscript) we do have cases for hummingbirds where camouflage is much needed and possible (given the predictability of the background pattern). For example, nesting females who are stationary indeed develop camouflaged patterns. Again, those do not constitute “ *ad hoc *explanations”, but a framework from which clear predictions can be made and that can be tested both in the laboratory and in the field.

 The reviewer mentions that “ *the figure appears to say that physiological constraints drive the environment, which is confusing to me*”. Such an inference would be confusing! However, the figure – and accompanying text – neither says nor implies this. The figure says “ *The “Possibility of camouflage” axis springs from the environmental and physiological constraints of the signal transmission between predators and prey*”. That is, animals can be physiologically constrained (e.g. given the species evolutionary history, the evolution of certain body colors might be impossible), but they can also be constrained due to the characteristics of the environment (as is the case of coral reef habitats for mobile animals).

 Still regarding the figure, the reviewer comments that “ *much more explanation is needed to allow the reader to understand how the different levels of analyses are included*”. The figure summarize and organize the ideas presented in the text under a theoretical framework, while attempting to be as visually-friendly and simple as possible (leaving the detail explanations for the body of the article). As such, the it risks being too dense, but I believe I succeeded in this task. In any case, I would be happy to answer and discuss any particular point that needs further clarification.

 The last observation of the reviewer “ *that the optics of the water as well as the visual system of the different viewers needs to be incorporated*” was addressed in the evolving online discussion in answers to previous reviewer comments, though not perhaps in the main manuscript. In any case, I am glad to add here another reference on this matter which was published after the hyper-visible world hypothesis was formulated, showing that coral reef fish can have excellent vision - in fact even better than human vision ( http://rsos.royalsocietypublishing.org/content/royopensci/3/9/160399.full.pdf). This observation adds empirical evidence to the challenges that animals -mainly mobile ones- have in concealing themselves in or against a backdrop of a coral reef environment.

# Peer review: Referee response for version 2

**DOI:** 10.5256/f1000research.8965.r14619

**Published:** 2016-06-28

**Authors:** Theodore Stankowich

**Affiliations:** 1 Department of Biological Sciences, California State University, Long Beach, CA, USA

In this article, the author introduces a predictive framework for understanding the bright coloration of coral reef fishes using the criteria of need for camouflage and possibility of camouflage. These axes allow fish to be grouped into fish that (1) able to adopt flamboyant colors because they don’t have significant predation due to some defensive ability, (2) live in a very visible colourful world and camouflage isn’t really possible anyway, and (3) adopt a cryptic coloration pattern due to great predation risk. I found this framework to be thought-provoking and apt for the tropical marine coral reef system under discussion.

 Every environmental medium has its own physical properties that promote or disfavour both organismal coloration and visual perceptual abilities. One issue with the article in its current form is that it could do a better job of discussing life on the margins of these systems where the light environment changes with depth – reds and oranges become dark browns and blues at greater depth and a discussion of this framework in a non-binary world would be interesting. Further, can brightly coloured fish “escape” their coloration temporarily at times of greater risk by moving into darker environments?

 The other discussion that would improve the piece is how it might differ in other media – how do physical differences between shallow marine environments differ with terrestrial environments in a way that allows this to occur. Clearly there are terrestrial environments the environmental background changes frequently or is spatially complex and animals live in a hyper-visible world – yet we see far fewer brightly coloured species on land, save for aposematic species (carefree world) and birds advertising for sexual display (sexual selection may be so strong as to override natural selection favouring camouflage). Why are there no brightly coloured mammals?  These questions are all easily addressed but would make for a more complete discussion of the hypothesis at hand and why it works in coral reef fishes.

 Overall, I found the paper thought provoking but additional discussion could make for a more complete, well-rounded discussion of the hypothesis and how it might even be adapted for other environmental systems.

I have read this submission. I believe that I have an appropriate level of expertise to confirm that it is of an acceptable scientific standard.

# Response

**Published:** 2016-07-29

**Authors:** Wladimir Alonso

**Affiliations:** Laboratory for Human Evolution Studies, Genetics and Evolutionary Biology, University of São Paulo, Brazil

Dear Theodore,

 Thank you very much for your comments. I am also glad you found the hypothesis presented in this paper to be thought-provoking and apt for the tropical marine coral reef system.

 Let me then address the specific points you rise:

1. “ **Every environmental medium has its own physical properties that promote or disfavour both organismal coloration and visual perceptual abilities. One issue with the article in its current form is that it could do a better job of discussing life on the margins of these systems where the light environment changes with depth**”. Indeed, the transitional zones are fascinating because the factors which favour the different (and often opposite) forces of selection can change in just a few meters. Those are all very interesting and relevant situations, and their complexity can actually be used to gather evidence for alternative hypotheses. For instance, the “hyper-visible world hypothesis” predicts that camouflage is not possible under good visibility and unpredictable backgrounds - conditions met for diurnal mobile fish of coral reef. As we move towards the margins of the system that you mention, lower lighting and/or less transparent waters would reduce the “signal to noise” ratio of mobile fish against the background, making cryptic coloration possible. In fact, this actually also happens during the diurnal cycle: the spectral complexity that corals display during the day slowly become less prominent when visibility start to dim at dawn. Natural experiments have the setback that many variables change simultaneously (fish in coral also start to lose the ability to detect visually approaching predators and to quickly hide in the coral interstices) so probably controlled experiments would be needed to falsify the alternative hypothesis. In any case, the intricacy of the transitional zones, as fascinating and deserving of study as they are, would demand a considerable expansion of the document. I thank, nonetheless, the opportunity to mention this aspect briefly here.
2. “ **can brightly coloured fish “escape” their coloration temporarily at times of greater risk by moving into darker environments?**”. That is an interesting question. Coral fish do move quickly inside corals and rocks interstices to escape from predators, but I believe they do it more due to the protection provided by the structural complexity than to become “invisible” in the dark.
3. “ **The other discussion that would improve the piece is how it might differ in other media – how do physical differences between shallow marine environments differ with terrestrial environments in a way that allows this to occur. Clearly there are terrestrial environments the environmental background changes frequently or is spatially complex and animals live in a hyper-visible world – yet we see far fewer brightly coloured species on land**...”. Hugh Cott, a zoologist who worked on military camouflage for the British Army during WWII, defended that this is the normal situation among birds: "few birds - whatever their coloration - can be expected to harmonize cryptically with surroundings which vary constantly and widely, from moment to moment and from month to month”. I agree only partially with Cott: although during flight is indeed difficult to come with a coloration that matches the changing background (or even the sky itself), many species expend a considerable time perched on branches or standing on the ground, and therefore cryptic coloration can be developed for those situations. Also, coral reef environments usually present a more diverse range of conspicuous colors (as opposed to mostly green and earth tones in terrestrial environments) due to the presence of pigments used to protect the symbiotic algae from high irradiances.
4. “... **save for aposematic species (carefree world) and birds advertising for sexual display (sexual selection may be so strong as to override natural selection favouring camouflage)**”.  I want to take advantage of this part of the sentence to highlight that the “carefree world” and the “hyper-visible world” do not lead necessarily to bright coloration. They lay the conditions whereby the selective pressure for camouflage (or, in different sensorial contexts, remaining in silence, being odorless, not moving, etc) is relaxed, allowing secondary functions for body coloration to emerge. So: (a)  aposematism can emerge either in the carefree or hypervisible context and (b) we can indeed assume that sexual selection is in general very strong, but I think it is also safe to assume that the use of coloration that favours mating is usually secondary to the need of not being seen either by predators or prey. That is why the suggested framework proposes that when we see bright coloration due to sexual selection (or aposematism, etc), it is interesting to ask whether this is because camouflage was not needed or was not possible (or both).
5. “ **Why are there no brightly coloured mammals?  These questions are all easily addressed but would make for a more complete discussion of the hypothesis at hand and why it works in coral reef fishes**”. Thank you again for the opportunity to discuss another interesting aspect that would be difficult to add in the paper due to space constraints. Firstly, your own studies show that, although rare, visual aposematism can be found in mammals - as in the case of the skunk’s bold stripes (to warn predators of the risk of being sprayed with stinky gas). Secondly, although bright coloration is indeed rare in mammals, they often announce vividly their presence with other sensorial channels: several carnivores (e.g our dogs) mark their territory with urine, monkeys do the same with sounds, many big terrestrial (and aquatic) mammals also don’t restrain themselves in vocalizing their presence (and using it for communication if needed). There is even a fascinating theory proposing that, by enabling early hominids to coordinate attacks (specially to steal food from lions), music itself was part of an “aposematic” route for human evolution (I warmly recommend to anyone interested in human evolution, aposematism or music to read the third chapter of Jordania’s book I cite in the article). So, although visual boldness is not so common in mammals, they are (if I may use the metaphor) “brightly colored” in other sensory channels (which usually have the advantage of being more easily “switched on and off” depending on the context, than body coloration). And finally I would argue again the earth-tones (sand, stones), snow and vegetation (mainly green and yellow/brown, resulting from chlorophyll and cellulose) of terrestrial environments are in fact quite predictable, and therefore more easily matched (at least at some extent) by some choice of body color/patterns (plus, terrestrial animals can indeed expend a considerable time still). All those conditions are in  radical opposition to the challenge faced by mobile fish in the colorful reef background.
6. “ **Overall, I found the paper thought provoking but additional discussion could make for a more complete, well-rounded discussion of the hypothesis and how it might even be adapted for other environmental systems**”. The paper started focusing on the formulation of a hypothesis to explain the conundrum posed by the abundance of bright colors fish in coral-reef, and later expanded to articulate a framework that could encompass the landscape of conditions that allow bright colors to emerge in any habitat. Therefore it is well possible that such broadness of scope could benefit from a lengthier document. Nevertheless, I hope that, caught in the compromise between comprehensiveness and suscinteness, I was able to lay out the ideas in a way that was both clear enough and stimulating for further research and discussions.

Thank you very much again for reviewing this paper and for your very relevant comments.

# Peer review: Referee response for version 1

**DOI:** 10.5256/f1000research.6967.r9330

**Published:** 2015-07-08

**Authors:** Brian Langerhans

**Affiliations:** 1 Department of Biological Sciences, North Carolina State University, Raleigh, NC, USA

The paper offers a hypothesis concerning the "striking colouration of coral reef fish." The idea is that coral reef fish roam across complex/unpredictable background colors and patterns within a transmission environment which permits highly effective perception of a range of light (color) to a variety of organisms capable of receiving and processing the respective light waves (during daytime). The argument rests on a number of assumptions, leading to the conclusion that selection will not favor camouflage in such an environment, and thus selection favoring conspicuous signaling is largely unconstrained. I think there is a useful hypothesis described here, which points out several testable ideas. However, I think each key assumption should be more directly addressed in the paper. Otherwise, one can see that the entire "Hyper-Visible World Hypothesis" could come crumbling down if those assumptions are wrong.

The first assumption needs more support: that coral reef fishes actually exhibit significantly higher color conspicuity or diversity than most other organismal assemblages. The entire hypothesis rests on this premise. Have studies tested whether coral reef fish actually exhibit such remarkable color patterns compared to other assemblages throughout the world? I agree that they surely must represent one of the more colorful, conspicuous, and diverse assemblages--but I'm not sure they are so remarkable that they deserve a special hypothesis unto themselves. It'd be nice to see some support for this premise. If this premise were untrue, then there is no need for a unique explanation for the colors of coral reef fishes.

The next assumption is that coral reefs provide a background of complex and unpredictable colors. I recall some studies showing low diversity of color during daytime and under ambient light for coral reefs. The author suggests that reefs are extremely colorful and complex, but I'd like to see some support for this. I agree they do seem fairly colorful and complex to us humans, but I can actually think of a number of other environments which seem pretty colorful and complex as well. What have previous studies found regarding the actual background colors of reefs? Are they really that complex and unpredictable from the perspective of a predator? Or is the background mostly blue/green/brown?

The next assumption is that coral reef waters actually are "hyper visible." That is, do these waters provide a transmission environment for which an especially wide range of color signals are accurately and efficiently transmitted? The waters are generally quite clear (low turbidity, high visibility), but air actually provides more effective transmission of light across all the relevant wavelengths. It seems to me that these waters actually truncate the red/yellow/orange wavelengths (to various extents), and thus are not "hyper visible" in that sense. Perhaps some further discussion of this point is warranted, as I'm not sure in what sense the waters are actually hyper-visible, but rather the organisms within the environment are highly visible due to a combination of water clarity and colors. 

The next assumption is that many resident organisms have well developed color vision. This is actually quite well supported by the literature, although it'd be nice for this paper to explicitly support this assumption as well.

The final assumption is really more of a prediction: that selection will not favor crypticity in such an environment, and thus we should not see cryptic organisms (but rather the evolution of conspicuous colors, which is the heart of the hypothesis). I doubt this is as universal as suggested in this paper. There are actually many examples of cryptic organisms inhabiting coral reefs (at least during a substantial part of their lives), including many fishes. Not only are there many coral reef fishes which are quite cryptic (e.g., peacock and leopard flounders, scorpionfish, frogfish, many blennies and gobies, trumpetfish...), but also many exhibit some cryptic components, such as countershading and disruptive coloration (including false eyes for misdirection). The prevalence of countershading seems to suggest that some crypticity has long continued to be advantageous in reef environments, although highly conspicuous signals are additionally advantageous. Moreover, the author's hypothesis that crypticity should primarily be favored in relatively sessile fishes could be directly tested (this could be mentioned in the paper)--and if the author could support this contention, that would be great (that most known examples of camouflage in reef fishes are in relatively stationary species).

I have read this submission. I believe that I have an appropriate level of expertise to confirm that it is of an acceptable scientific standard, however I have significant reservations, as outlined above.

# Response

**Published:** 2016-03-07

**Authors:** Wladimir Alonso

**Affiliations:** Laboratory for Human Evolution Studies, Genetics and Evolutionary Biology, University of São Paulo, Brazil

Dear Brian,

Thank you very much for your review, relevant comments and for allowing me the opportunity to provide more evidence and clarify the assumptions on which my proposed hypothesis rests on. Your name, together with others –who provided me with feedback, will be included in the acknowledgment section of this manuscript, in recognition of your time and expert feedback. In totality, the constructive criticism and discussion this paper has generated has helped me realize that I should also somehow present these ideas in a systematized and slightly more organized format. The current version therefore provides a framework that more formally addresses the topic of the conspicuous coloration of certain animal species, like macaws and hummingbirds, which were just included as an additional note in previous version. Without adding too much extra content, I managed to create a couple of new sections including a new designation - "the carefree world" in addition to a figure that both integrates and summarizes those ideas.

Below I have addressed the assumptions you specifically mentioned and that would need further support and clarification:

#1 " ***coral reef fishes actually exhibit significantly higher color conspicuity or diversity than most other organismal assemblages*** *. The entire hypothesis rests on this premise. Have studies tested whether coral reef fish actually exhibit such remarkable color patterns compared to other assemblages throughout the world?*"

**WJA Reply:**

I haven't succeeded in finding a reference of a study that quantifies that coral reef fish exhibit higher colour conspicuity of diversity than most other organismal assemblages. It seems that it is a given in the field, and scientists express in sorts of ways like this:

"There is in all the world, no other biotope which has produce, in so short a time or, which means the same thing, in so closely allied groups of animals, an equal number of extremely specialized forms" (1) or  " *A coral reef is perhaps the most colorful of all the world's ecosystems. During the day it throngs with multicoloured fish and invertebrates*" and "there is nowhere on earth where one can find so many colourful animals packed into such small space" (2) Nevertheless, despite perhaps not being quantified in the way we would like it, this is an observation that is widely supported and the one which all the hypothesis mentioned in this text since Wallace's one, try to find an answer.

Furthermore, allow me to also address your remark that " *I agree that they surely must represent one of the more colorful, conspicuous, and diverse assemblages--but I'm not sure they are so remarkable that they deserve a special hypothesis unto themselves. It'd be nice to see some support for this premise. If this premise were untrue, then there is no need for a unique explanation for the colors of coral reef fishes*".

**WJA Reply:** As surprising as it may seem, the abundance of bright and colourful fish in coral reef , that so markedly stands out visually, -where we should expect to witness animals trying to blend into the surroundings, so they do not become “an obvious meal”(3) is still an open question and the subject itself, has given rise to several studies and conjectures to address it (as shown in the introduction of the paper). I do agree with you on your point that any hypothesis looking at it should aim for a broader scope, as mechanisms in nature rarely are specific to only one circumstance. I just found a passage in Cott's 1940 book that can be considered another example of a "hyper-visible world," which I am including as a opening citation in the "Hyper-visible world" section of the new version

#2 " ***coral reefs provide a background of complex and unpredictable colors*** *. I recall some studies showing low diversity of color during daytime and under ambient light for coral reefs. The author suggests that reefs are extremely colorful and complex, but I'd like to see some support for this. I agree they do seem fairly colorful and complex to us humans, but I can actually think of a number of other environments which seem pretty colorful and complex as well. What have previous studies found regarding the actual background colors of reefs? Are they really that complex and unpredictable from the perspective of a predator? Or is the background mostly blue/green/brown?*

**WJA Reply:** Matz et al (4) used spectrometry data and visual system modelling to answer the question posed in the title of their article "Are Corals Colourful?" This article attempted to answer such questions from the perspective of the fish and found that " *Some GFP-like proteins, most notably fluorescent greens and nonfluorescent chromoproteins, indeed generate intense color signals*."  They go on to explain that " *fluorescent proteins might also make corals appear less colorful to fish, counterbalancing the effect of absorption by the photosynthetic pigments of the endosymbiotic algae, which might be a form of protection against herbivores.*" But as they even discuss at the end,  " *It is tempting to speculate that the evolution of diverse coral colors in early Mesozoic (3) might have prompted the specialization of the fish visual systems in the process of adaptation to the environment, eventually leading to appearance of very unusual color signaling displayed by present-day reef fishes.*" Therefore, I believe it is a sound assumption that coral reefs are not only perceived as colourful to humans but are also perceived as such by (often chromatically more sophisticated eyes (5)) their aquatic inhabitants as well. I modified the text accordingly to include these references and a summary of those considerations.

In any case, please note that if colour vision were not found in predatory interactions within the context of the hypothesis presented, this would not be an issue for the hypothesis since mobile fish in the "hyper-visible world" cannot hide, regardless of their colour, meaning predators/prey also need not use colour vision (again, for their interactions). This is also valid in the "carefree world" scenario, where, if a predator cannot risk hunting a dazzlingly colourful macaw, why would evolution bother providing that predator with the visual capabilities customized to detect a macaw?

# 3 " **coral reef waters actually are "hyper visible.**" That is, do these waters provide a transmission environment for which an especially wide range of color signals are accurately and efficiently transmitted? The waters are generally quite clear (low turbidity, high visibility), but air actually provides more effective transmission of light across all the relevant wavelengths. It seems to me that these waters actually truncate the red/yellow/orange wavelengths (to various extents), and thus are not "hyper visible" in that sense. Perhaps some further discussion of this point is warranted, as I'm not sure in what sense the waters are actually hyper-visible, but rather the organisms within the environment are highly visible due to a combination of water clarity and colors"

**WJA Reply:**  Coral reefs are usually found in shallow, clear tropical waters where the spectral attenuation  of light by transmission medium (water) has not  yet been severe.  (2). In fact, only very little of the downwelling light, while the horizontal light is "less intense, narrower and shifted to the blue" (5)(6). Many fish can see even UV light, so they are able to perceive an even greater variety of colours than we do. You are correct in pointing out (and I do also points this out in the paper) that the excellent visibility of coral reefs habitats are nonetheless inferior to most of the terrestrial. But do note that the "hyper-visible world" is a way to express the impossibility of matching a background pattern in certain conditions and that difficulty is imposed not only because of the good transparency and luminosity of the medium, but equally important is the unpredictability of the background to be matched. Terrestrial environments (as discussed in the first answer) seem to be more predictable (but, again, please see the first new paragraph of the Hyper-visible World section quoting an interesting parallel explanation in Cott's 1940 book) . While this is true for visual signals, perhaps this is not the case for acoustic and olfactory signals (which is why I added that small discussion at the end of the paper in the current version.

# 4 " ***many resident organisms have well developed color vision*** *. This is actually quite well supported by the literature, although it'd be nice for this paper to explicitly support this assumption as well*"

**WJA Reply:** As suggested, I added Bennet et al review (7), which explains how vision in those environments can be more sophisticated than ours (including UV vision and a higher number of different photoreceptors, which  are common in marine species.

# 5 " *The final assumption is really more of a prediction: that **selection will not favor crypticity in such an environment, and thus we should not see cryptic organisms (but rather the evolution of conspicuous colors, which is the heart of the hypothesis)**. I doubt this is as universal as suggested in this paper. There are actually many examples of cryptic organisms inhabiting coral reefs (at least during a substantial part of their lives), including many fishes. Not only are there many coral reef fishes which are quite cryptic (e.g., peacock and leopard flounders, scorpionfish, frogfish, many blennies and gobies, trumpetfish...), but also many exhibit some cryptic components, such as countershading and disruptive coloration (including false eyes for misdirection).*"

**WJA Reply:**  You spotted an error that was also pointed out by a comment from Benjamin Geffroy; that is,  I incorrectly used the expression "will" where it should read "can." I have since  corrected it in the revised version. But please do note that this hypothesis stresses the importance of the "mobile" (i.e. spatio-temporal) feature for making it very difficult for organisms to evolutionary develop a pattern that can provide some concealment against predators or preys. I hope that the new figure added to the paper helps to further clarify the circumstances where coral reefs can indeed support camouflage (which basically occurs when animals are loyal to one specific background during daylight hours).

*#6 "The prevalence of countershading seems to suggest that some crypticity has long continued to be advantageous in reef environments, although highly conspicuous signals are additionally advantageous. Moreover, the author's hypothesis that crypticity should primarily be favored in relatively sessile fishes could be directly tested (this could be mentioned in the paper)--and if the author could support this contention, that would be great (that most known examples of camouflage in reef fishes are in relatively stationary species)".*

**WJA Reply:** Indeed, countershading, disruptive camouflage and other forms of concealment are not negated among diurnal mobile fish in coral reefs. The extraordinary abundance of life forms and solutions to the constant predator/prey interactions certainly include these mechanisms (and possibly others we haven't described yet). Nevertheless, the question remains of why there are so many fish that are so easily detectable, even for humans. Furthermore, this species is only a visitor in the described environments and the reason for why senses which do not define the need to see those species - is not covered by those explanations. It has been shown that fish can develop visual capabilities that are far better than ours - if doing so would provide them with the ability to escape from or catch those colourful fish, it would be surprising that they would not use/develop those very capabilities.

Thank you once again for your appreciated input.

Best,

Wladimir

1.     Lorenz K. The function of colour in coral reef fishes. Proc Roy Inst Gt Brit. 1962;39:282–96.

2.     Marshall JJ. The visual ecology of reef fish colours. Animal signals: signaling and signals design in animal communication In: Espmark, Y, Amundsen, D, and Rosenqvist, G (eds) Animal signals: signaling and signals design in animal communication. Norway: Tapir Academic Press; 1999.

3.     Marshall J. Pacific Ocean: Why Are Reef Fish So Colorful? Sci Am Oceans. 1998;August.

4.     Matz MV, Marshall NJ, Vorobyev M. Are Corals Colorful? Photochem Photobiol. 2006 Mar 1;82(2):345–50.

5.     McFarland WN, Munz FW. Part III: The evolution of photopic visual pigments in fishes. Vision Res. 1975 Oct;15:1071–80.

6.     McFarland WN, Munz FW. Part II: The photic environment of clear tropical seas during the day. Vision Res. 1975 Oct;15:1063–70.

7.     Bennett ATD, Cuthill IC, Norris KJ. Sexual Selection and the Mismeasure of Color. Am Nat. 1994 Nov 1;144(5):848–60.
